# *Helicobacter Pylori* Infection Induces Intestinal Dysbiosis That Could Be Related to the Onset of Atherosclerosis

**DOI:** 10.1155/2022/9943158

**Published:** 2022-10-22

**Authors:** Avilés-Jiménez Francisco

**Affiliations:** Unidad de Investigación Médica en Enfermedades Infecciosas y Parasitarias, UMAE Pediatría. Centro Médico Nacional Siglo XXI. IMSS, Ciudad de México, Mexico

## Abstract

Cardiovascular diseases represent one of the first causes of death around the world, and atherosclerosis is one of the first steps in the development of them. Although these problems occur mainly in elderly, the incidence in younger people is being reported, and an undetermined portion of patients without the classic risk factors develop subclinical atherosclerosis at earlier stages of life. Recently, both the *H. pylori* infection and the intestinal microbiota have been linked to atherosclerosis. The mechanisms behind those associations are poorly understood, but some of the proposed explanations are (a) the effect of the chronic systemic inflammation induced by *H. pylori*, (b) a direct action over the endothelial cells by the cytotoxin associated gene A protein, and (c) alterations of the lipid metabolism and endothelial dysfunction induced by *H. pylori* infection. Regarding the microbiota, several studies show that induction of atherosclerosis is related to high levels of Trimethylamine N-oxide. In this review, we present the information published about the effects of *H. pylori* over the intestinal microbiota and their relationship with atherosclerosis and propose a hypothesis to explain the nature of these associations. If *H. pylori* contributes to atherosclerosis, then interventions for eradication and restoration of the gut microbiota at early stages could represent a way to prevent disease progression.

## 1. Introduction

Cardiovascular diseases (CVDs) are a group of heart and blood vessels disorders, responsible for disability and premature mortality that include ischemic heart disease (IHD), coronary artery disease, cerebrovascular events (strokes), high blood pressure, and blockage of different vessels of the circulatory system, and they represent one of the first causes of death globally. According to the World Health Organization, 17.9 million people die every year due to CVDs, and around 75% of all deaths occur in low-income countries. Although these problems occur mainly in the elderly, there is some incidence in younger people [1]. The recognized risk factors for CVDs are hypertension, diabetes, hyperlipidemia, obesity, smoking, physical inactivity, and stress conditions as well as some genetic factors associated to an increased risk of CVDs [2–7]. However, not all cases present these factors and an undetermined portion of individuals develop symptoms early in life [8]. Under this scenario, it is relevant to study the role and possible interactions between these and new factors like the microbiota and bacterial infections to gain knowledge of the mechanisms that govern the establishment of CVDs.

### 1.1. The Initial Step in the CVDs Is the Development of Atherosclerosis

Atherosclerosis is a chronic inflammatory process characterized by lipid, fibrous tissue, and cellular components accumulation on the arterial cell wall, forming a plaque called atheroma that disorganizes the architecture, alters the vascular function, and reduces the heart blood flow. Several components of the inflammatory response including peripheral blood mononuclear cells and macrophages participate in the initial damage. They respond to an increased expression of cellular adhesion molecules like *β*2 and *β*1-integrins, the E-selectin and P-selectin ligands, together with an increased endothelial permeability. Macrophages transform into foam cells by capture of modified low-density lipoproteins (LDL) and smooth muscle cells migrate towards the atheroma. These processes reduce the blood vessel lumen causing ischemia, and if a subsequent erosion or plaque rupture happens, a thrombotic event might result with fatal consequences. There are two possible initiators of an atheroma, a lesion in the vascular system due to a chemical compound or the immune response against an infectious agent [9]. In the initial steps of *H. pylori* infection, there are subclinical inflammation and metabolic disorders, and the same conditions are also observed in atherosclerosis. These similarities rise the question, is *H. pylori* infection associated with atherosclerosis through a common link or process? One hypothesis for this problem is that the common link is changes in the intestinal microbiota, which have been widely related to both pathologies.

## 2. Microbiota and Atherosclerosis

The term “microbiota” includes all the microorganisms (prokaryotes, archaea, protozoa, fungi, and viruses) that populate the human body, while the term “microbiome” refers to a characteristic microbial community occupying a reasonable well-defined habitat and their corresponding set of genes and genomes as well as the products of the microbiota and the host environment [10–13]. The human microbiota performs functions which are essential for health maintenance, involving the immune system activity and energy homeostasis. The microbial composition is a signature for each body habitat; however, the diversity and abundance vary within and among individuals. The gut microbiota is home to the largest number of species, and consequently, the number of metabolic processes is greater compared to other regions of the body. The phyla *Firmicutes* and *Bacteroidetes* account for >98% of all species, and the rest is distributed among *Actinobacteria*, *Fusobacteria*, *Proteobacteria*, *Verrucomicrobia*, and *Cyanobacteria*, whose proportions remain relatively stable in time [14]. When these proportions are altered, a state of dysbiosis is produced which is characterized by diversity loss, changes in the type and quantity of organisms and the metabolic processes [15]. In this review, the term microbiota will be used to refer to the intestinal microbiota, which is the main interest of this work.

### 2.1. Dysbiosis Is Associated with Atherosclerosis and CVDs

In recent years, the role of microbiota in relation to CVDs has been the objective of extensive analysis (Table 1). Some hypotheses have been formulated to explain the nature of these associations. One of them propose that lipopolysaccharide (LPS) from Gram-negative bacteria induces a low-grade systemic inflammation, promoting CVD by activation of Toll-like receptor 4, and the expression of proinflammatory mediators such as IFN*γ*, IL-1, IL-6, IL-8, and TNF [16]. LPS is also considered a proatherogenic mediator, as it promotes lipid accumulation in adventitial fibroblasts [17]. Moreover, LPS induces foam cell formation from macrophages by inhibiting the cholesterol efflux. This mechanism is possibly mediated by the adipocyte enhancer-binding protein 1 (AEBP1) as well as alterations in the CD36 receptor and the ATP-binding cassette transporter A1 [18, 19].

Previous descriptive microbiome analyses indicate that members of *Erysipelotrichaceae* and *Lachnospiraceae* families correlated with disease markers (total cholesterol and LDL cholesterol levels) [20]. In relation to metabolic pathways, the genes responsible for peptidoglycan synthesis were enriched in atherosclerosis patients while the phytoene dehydrogenase which is responsible for the lipid-soluble antioxidants metabolism were enriched in healthy individuals [21]. Similarly, in a larger metagenomic study, a higher abundance of *Enterobacteriaceae* and *Streptococcus* spp, in combination with a lower abundance of butyrate-producing bacteria (*Bacteroides* spp., Prevotella copri, and *Alistipes shahii*) were reported in CVD patients. [22]. Similar data were obtained in the microbiome analysis of patients with carotid atherosclerosis. The enrichment of 21 species was reported, highlighting the presence of *Bacteroides eggerthii*, *Escherichia coli* and *Klebsiella pneumoniae*. On the contrary, unclassified *Parabacteroides*, *Prevotella copri*, *Bacteroides sp* 3_1_19 and *Haemophilus parainfluenzae* appeared with greater abundance in healthy individuals. In addition, from 142 identified metabolic pathways, those related to elevated levels of Trimethylamine N-oxide (TMAO) are distinguished [23]. Advances in metagenomics have allowed the identification of various favorable and unfavorable species for health, so the use of the microbiome as a biomarker of cardiometabolic risk has recently been proposed [24]. Furthermore, with the help of artificial intelligence through the discipline of machine learning, a microbiome-based diagnostic test for CVD was developed. This test considers that a core set of the microbiota represents a common denominator for the entire clinical spectrum, and uses 39 taxonomic groups that clearly differentiate subjects with or without CVD [25].

In addition to all the information generated from CVDs patients, the finding that subjects with SCA also develop intestinal dysbiosis is notorious. In these individuals, there is an increased abundance of *E. coli* and members of the *Streptococcus* genus. In contrast, member of the *Bacteroides* genus and *Faecalibacterium prausnitzii* are more abundant among healthy controls. These findings raise the question about the possibility to develop an early diagnostic test when there are no CVD symptoms yet [26].

### 2.2. The Microbiota Metabolism Has a Significant Effect in Host Physiology Related to Atherosclerosis

Microbiome studies have shown that bacterial metabolism is perhaps more relevant than description of the taxonomic groups inhabiting the gut, and the changes in metabolic activity obey to environmental factors like the available nutrients [27]. For CVDs, dysbiosis is responsible in part of modifying some of the relevant risk factors, such as obesity [28–32], type II diabetes [33, 34], and inflammation [35]. From all studied metabolites, TMAO has attracted special attention since higher plasma levels were associated with an increased risk of atherosclerosis both in humans and mice [36]. TMAO is the final product of choline, phosphatidylcholine, L-carnitine, and *γ*-butyrobetaine metabolism, and the main source is consumption meat, fish, and eggs. The intermediate product trimethylamine (TMA) can also be produced by an endogenous biosynthetic pathway using lysine as substrate to form *γ*-butyrobetaine [37, 38]. It is still unknown if any other bacterial system has the capability to liberate TMA from diet. The first step in TMAO formation is the generation of TMA by the action of the microbial enzyme complexes (a) CutC/D (the choline utilization TMA lyase system), (b) the CntA/B, or (c) the YeaW/X (carnitine Rieske-type oxygenase/reductase system) [38–41]. These systems are present in the *Firmicutes*, *Proteobacteria*, *Actinobacteria*, and *Fusobacteria phyla* but absent in the Bacteroidetes group. Some of the bacterial genera reported with these systems include *Desulfovibrio, Clostridium, Streptococcus, Klebsiella, Enterobacter, Escherichia, Anaerococcus, Aeromonas, Proteus, Providencia, Vibrio, Collinsella, Desulfotomaculum, Desulfotalea, Desulfosporosinus, Desulfitobacterium, Alkaliphilus, Olsenella, Paenibacillus*, and *Yokenella* [42]. The CutC gene cluster has also been identified in the oral microbiome (supragingival plaque, buccal mucosa, and tongue), which is relevant as periodontal disease and some bacterial species have been associated with atherosclerosis and CVD [43]. Once produced, TMA is absorbed and transported through the blood flow into the liver where it is oxidized by the flavin monooxigenasa3 (FMO3) to form TMAO. The FMO3 activity is regulated by bile acids, and their effect on the gene expression is mediated by activation of the nuclear hormone receptor foresaid X FXR (NR1H4) [44]. As flavin-monooxygenases are also present in the marine prokaryotic kingdom, it is thought that TMAO is possibly produced by the intestinal microbiota itself [45].

As higher TMAO levels are described as a potential inductor of endothelial damage and possibly a proatherogenic factor in humans, the role of the genetic components associated with plasma TMAO levels needs to be investigated. In this regard, a comparative Genome-Wide Association Study looked for loci related to plasma TMAO levels, and the results indicated that the genetic component is not that relevant. Instead, it was reported that variations in diet or microbiome are more important than genes in determining the plasma TMAO levels [46].

### 2.3. Higher TMAO Concentration Correlate with an Increased Risk of Cardiovascular Events

The role of TMAO in CVDs has been remarked by the inhibition of TMA formation by 3,3-dimethyl-1-butanol (DMB). This action reduces the TMAO levels and in consequence inhibits the macrophage foam cell formation, platelet responsiveness, and thrombus formation [47, 48]. In different studies, TMAO levels correlate positively with an increased risk for the incidence of major adverse cardiovascular events [49, 50], increased mortality in patients with a history of heart failure [51], and with acute coronary syndrome [52]. In a recent meta-analysis from prospective studies including 10,301 individuals with coronary heart disease, higher TMAO levels increased in 58% the risk to suffer a major adverse cardiovascular event. In this study, the authors established a concentration of 5.1 *μ*mol/L as the cut-off value for prognosis [53]. In contrast, some authors argue that plasma levels of TMAO can serve as a predictor for plaque instability rather than an indicator for the extent of atherosclerosis [54].

The mechanism by which TMAO induces atherosclerosis is still poorly understood. One explanation proposes that TMAO promotes macrophage cholesterol uptake by increasing the expression of scavenger receptors such as CD36 and SRA1 [36], as well as the inhibition of the reverse cholesterol transport pathway and alteration of bile acid metabolism [37]. Another proposal is based on the fact that microbiota seem to modulate the platelet hyperresponsiveness and clot formation by TMAO generation and the release of intracellular calcium in platelets [55, 56]. An alternative explanation considers the direct action over endothelial cells and smooth muscle cells, inducing endothelial dysfunction and inflammation. The inflammatory response comprises increased macrophage recruitment and increased expression of adhesion molecules. Among the adhesion molecules with the higher expression are monocyte chemotactic protein 1 (MCP-1), macrophage inflammatory protein 2 (MIP-2), tumor necrosis factor-*α* (TNF-*α*), intercellular adhesion molecule 1 (ICAM1), cyclooxygenase 2 (COX2), E-selectin, vascular cell adhesion molecule 1 (VCAM-1), and the CD36 receptor. However, until now no specific TMAO receptor has been identified in endothelial cells [57–59]. Another proposed mechanism implicates the induction of NLRP3 (NOD-like receptor Pyrin domain containing) inflammasome as its activation is critical in foam cell formation and represents the initial step for endothelial dysfunction. This process possibly involves the activation of thioredoxin-interactive protein linked to NLRP3 signaling, via reactive oxygen species (ROS-TXNIP-NLRP3) and the tight-junctions disruption and alteration of endothelial cells permeability leading atherosclerosis [60, 61].

However, the role of higher TMAO levels in atherosclerosis is still controversial, as a diet supplemented with TMAO did not induce atherosclerosis in a mouse model [62]. This suggests that elevated TMAO is just an indicator of dysbiosis rather than a cause for atherosclerosis [63]. Even some authors consider that moderately elevated TMAO is potentially beneficial in cases of pressure-overloaded heart [64]. In agreement with this idea some studies suggest that TMA is responsible for the induction of atherosclerosis, as it exerts a cytotoxic effect on cardiomyocyte [65]; it shows hemodynamic effects in rats [66], and affects the viability of human vascular smooth muscle cells [67]. A more detailed analysis of the mechanisms of action of TMA/TMAO in atherosclerosis is necessary.

## 3. *Helicobacter pylori* Infection and Atherosclerosis

*H. pylori* is a Gram-negative gastric pathogen that colonizes more than half of the human population inducing chronic active gastritis, peptic ulcer disease, mucosa associated lymphoid tissue lymphoma, and gastric adenocarcinoma. The infection is generally acquired during childhood and may last for decades if not treated. It is characterized by a strong inflammatory process and induction of an active immune response. In response to the infection, cells from the immune system are attracted to the lamina propria by the effect of chemokines like IL-8, monocyte chemoattractant protein-1, growth related oncogene-*α*, and IL-1*β*. These chemokines are produced and secreted by gastric epithelial cells in response to the action of bacterial virulence factors like the vacuolating cytotoxin (VacA), CagA protein, and urease. At the gastric mucosa, the immunocompetent cells are activated and exert their effector functions such as the production of cytokines (IL-1*β*, TNF-*α*, IL-6, IL-12, and IFN-*γ*), chemokines (IL-8 and MCP-1), nitric oxide (NO), and reactive oxygen species (ROS). If the infection is not resolved, the process is perpetuated evolving into a chronic inflammatory state [68]. The association between *H. pylori* and atherosclerosis is not clearly understood, but it is possible that the chronic inflammatory process induced by the bacterium is an indirect mechanism of endothelial cells damage, favoring the development of atherosclerosis [69, 70].

In 1994, Mendall et al. published the first report about an association between seropositivity to *H. pylori* and coronary heart disease [71]. Since then, multiple studies have investigated this observation, but the underlying mechanisms behind this phenomenon are not known (Table 2). The hypothesis that *H. pylori* has a role in the induction of atherosclerosis is based on the association between anti-*H. pylori* IgG antibodies and adverse cardiovascular disease, arterial stiffness, coronary artery calcium, and subclinical artery stenosis [72–76]. In addition, DNA detection and the successful *H. pylori* isolation from atherosclerotic plaques support the idea of a direct action on the endothelial surface [77–79]. Moreover, this association has also been analyzed in healthy individuals by computed tomography, and the incidence of SCA was higher in *H. pylori* asymptomatic carriers (OR 2.813, 95% confidence interval 1.051 ± 7.528, *P* = 0.04) [80]. But not only the bacterial infection is associated, the presence of antibodies against the CagA virulence factor has also been associated with aortic atherosclerosis and acute coronary events. The CagA protein is the most studied *H. pylori* virulence factor which is encoded by the *cag* pathogenicity island (*cag*PAI), and it is present in approximately 60-80% of clinical isolates [81, 82]. One possible explanation for this association is the cross-reaction between the anti-CagA antibodies with epitopes located in the arterial wall [83–86]. On the other hand, it has also been described the presence of extracellular vesicles (exosomes) containing CagA in the serum of *H. pylori* infected patients, and even gastric epithelial cells expressing CagA *in vitro* show the secretion of active CagA-containing exosomes [87]. In animal models infected with *H. pylori*, CagA-containing exosomes derived from gastric epithelial cells promote macrophage-derived foam cell formation and downregulate the expression of cholesterol efflux transporters [88]. In agreement with these reports, Xia et al. showed that an exosome-mediated mechanism induces endothelial dysfunction in a mouse model and in patients infected with cagA-positive strains [89]. More recently, it was found that CagA inhibits the LDL uptake into cells by interacting with the low-density lipoprotein receptor (LDLR) via its C-ter region. Ninomiya et al. hypothesized that this could represent an alternative mechanism by which cagA+ strains lead to hypercholesterolemia that is recognized as a classical risk factor for induction of atherosclerosis [90].

Another explanation for the *H. pylori*-atherosclerosis association considers the induction of endothelial dysfunction by elevated levels of homocysteine. It is known that *H. pylori* gastritis decrease the absorption of vitamin B12 and folic acid and increase the levels of homocysteine and C-reactive protein, which possibly mean the initial step of atherosclerotic plaque formation [91, 92].

Finally, in a recent meta-analysis about the infection prevalence in patients with adverse cardiovascular events that included 40 studies published between January 1, 1990 and January 31, 2020 (*n* = 19,691), it was concluded that *H. pylori* infection increases the risk of developing atherosclerosis and CVD by 51% [93].

## 4. *Helicobacter pylori* Infection and Intestinal Microbiota

Colonization of the gastric mucosa by *H. pylori* not only alters the local ecosystem, but it also may alter the microbial composition at the lower intestinal tract, and in consequence, modulate the concentration of microbial metabolites that can be used as disease markers. Different studies have analyzed the relationship between *H. pylori* and the gut microbiota (Table 3). Some data supporting this idea came from the metabolome of peptic ulcer disease patients due to *H. pylori*. In this analysis, the long-chain fatty acids blood profile appears to be disease-specific, and the authors argue that this remarkable change in the metabolic profile cannot be explained by the presence of a single pathogen in the stomach but by substantial changes in the microbial community at the lower gastrointestinal tract [94]. In infected gerbils, the microbiota analysis shows important changes in the distribution and bacterial density in both the stomach and the duodenum, and in the C57Bl/6 murine model, similar alterations were observed in either the gastric mucosa and the distal gut [95, 96]. In a similar way, *H. pylori* infection seems to modulate the microbial communities in humans. In infected children (6-12 years old), there were significant microbiota changes in comparison to noninfected controls, suggesting that microbial alterations could even be induced at early stages of life [97, 98]. Among the bacterial groups with significant changes in diversity and abundance due to *H. pylori* infection are the phylum *Bacteroidetes*, *Firmicutes*, and *Proteobacteria*. Particularly, there is a notorious decrease in the relative abundance of *Bacteroidetes*, while the *Firmicutes* are increased [99, 100]. At the genera level, the *Lactobacillus* group seems to experiment important changes in their relative abundance. While *Lactobacillus salivarious* experiments, an increased abundance in infected patients, *Lactobacillus acidophilus* is more abundant in noninfected subjects [101]. There is even data suggesting that *H. pylori* influences the composition of fungal populations in the intestinal tract [102]. Apart from the microbiota changes, *H. pylori* is associated with alteration in the microbial metabolism. In a Chinese population study, a reduction in the vitamin B12 biosynthetic module and decreased plasma vitamin B12 concentration was observed in *H. pylori* positive patients [103]. These data agree with previous studies about lower plasma vitamin B12 levels in *H. pylori* patients, but they also offer a potential explanation to the fact that vitamin B12 deficiency relates to SCA and correlates with the categories of coronary artery disease [104–106].

Another evidence about the altered microbial metabolism due to the infection is the different patterns of fecal fatty acids between *H. pylori* infected and noninfected individuals and the altered bacterial diversity. However, in infected patients some specific taxa are enriched (*Atopobium*, *Gemellaceae*, *Micrococcaceae*, *Gemellales*, and *Rothia*). It is interesting to note that those enriched taxa belong to the phylum *Firmicutes* and *Actinobacteria*, which are reported to possess the metabolic capability to produce TMA [107].

Very few information about a possible interaction between *H. pylori* and the microbial metabolite TMAO is available. Experimental infection of gastric epithelial cells and exposed to TMAO showed a synergistic effect on the expression levels of immunoinflammatory response genes such as IL-6, CXCL1, CXCL2, FOS, and C3. It is not known if this interaction actually takes place *in vivo*, but if so, it would be important to consider the effects of both conditions to determine their role whether in gastritis or in atherosclerosis development [108]. In a mouse model, choline supplementation of infected mice increased the serum TMAO levels and elevated the concentration of inflammatory components (C reactive protein, LPS, IL-6, TNF-*α*, and CXCL1). These components may exacerbate the *H. pylori* induced inflammation and modify the richness and diversity of the microbiota, particularly the relative abundance of the Escherichia-Shigella group [109].

### 4.1. The Inflammation Induced by *H. pylori* Raises a Healing Response That May Have an Effect on the Microbiota

The immune response to *H. pylori* infection is characterized by infiltration of neutrophils and specific immune cells and the release of reactive oxygen species (ROS), such as superoxide anion (·O2–), peroxide hydrogen (H2O2), and the hydroxyl radical (·OH–). To compensate the oxidative stress, different mechanisms are activated in order to protect the integrity of the mucosa which includes the release of antioxidant enzymes like catalase, superoxide dismutase and glutathione as well as other endogenous antioxidant compounds such as ghrelin and L-carnitine [110].

L-carnitine (L-3-hydroxy-4-N, N, and N-trimethylaminobutyrate) is a biomolecule involved in the transport of long chain fatty acids from the cytoplasm to the mitochondrial matrix where *β*-oxidation takes place. It is also an antioxidant that protects the gastric mucosa by inhibition of the xanthine-oxidase enzyme [111, 112], capture of free radicals [113–116], reduction of neutrophil accumulation [117], protection from peroxide damage [118–120], and inhibition of gastric mucosa damage from ethanol [121]. In mammals, its main source is the food proteins degradation, but it can be synthesized endogenously in the liver and the kidneys from the aminoacids lysine and methionine [122]. However, synthesis de novo has also been reported to take place in the gut of a mouse model [123]. As L-carnitine is also a precursor for TMA, it is not known if these biosynthetic routes are relevant for the TMA cycle that contributes for atherosclerosis development. In particular, *H. pylori* infection, which in many cases lasts for decades, represents a constant stimulus for the synthesis of L-carnitine in the gastrointestinal tract [49, 124].

A second compound involved in the gastric anti-inflammatory response is Gastrokine-1 (GKN1). It is a protein expressed in the superficial/foveolar gastric epithelium (cardia, body, and antrum). GKN1 confers protection and promotes the healing of mucosal lesions by favoring the proliferation and restitution of epithelial cells, and it seems to play a role in suppression of the carcinogenic process [125, 126]. GKN1 is made up of 185 amino acids including an N-terminal signal peptide, and it has a characteristic central domain called the BRICHOS domain, which might be involved in disulfide bridges [127–129]. In *H. pylori* infection, GKN1 expression is downregulated depending on disease severity, so it was thought that GKN1 was completely abolished in gastric cancer cases [130]. However, the expression is not completely suppressed as it is still detected in patients without cancer or even in cancer patients but in nontumoral tissues. These findings possibly represent a tissue repair process and at the same time a tumor containment mechanism [131–133]. Epidemiological studies indicate that between 1-3% of infected people develop cancer, so the lasting effect of GKN1 expression on the rest of the cancer-free population is unknown.

It is thought that the downregulation mechanism involves the activation of the ERK pathway by the CagA virulence factor, promoting the upregulation of the AU-rich element RNA-binding factor 1 (AUF1), that negatively regulates the GKN1 expression [132]. If CagA downregulates GKN1, then the infection with CagA negative strains is a relevant aspect, as it is estimated that between 20-40% of the clinical isolates are *cagA* negative. On the other hand, some authors propose that GKN1 possibly suppresses the CagA-induced effects on epithelial cells avoiding the gastric cancer progression [134].

In a recent study using a GKN1^−/−^ mouse model, it was reported that GKN1 indirectly mediates obesity through its effects on the gut microbiota. GKN1 was detected not just in the stomach but also in the small bowel and in the colon. GKN1^−/−^ mice exhibited an altered gut microbiota with significant reduced prevalence of *Firmicutes*, which was translated in resistance to diet induced obesity in comparison to wild type mice [135]. These findings are consistent with previous reports in which an increased abundance of Firmicutes is associated with obesity in both humans and mice [29, 30, 136]. The fact that GKN1 modifies the microbiota to modulate obesity is relevant as obesity is a well-recognized risk factor for CVDs. Moreover, members of the phylum *Firmicutes* have the metabolic ability to produce TMA/TMAO which are strongly associated with atherosclerosis. The precise mechanism by which GKN1 affects the microbiota is not known, but one possibility is the action of its antiamyloidogenic properties to avoid polypeptides aggregation and prevents biofilm formation [137–139]. In the gastrointestinal tract, biofilm formation is a process related to gut homeostasis and disease, and if GKN1 prevents biofilm formation, then the homeostasis and the microbiota equilibrium will be altered [140, 141]. Another possible mechanism is the participation of the BRICHOS domain that seems to be involved in the biosynthesis of antimicrobial peptides [142, 143]. The role of GKN1 in the induction of dysbiosis needs to be further investigated.

### 4.2. *H. pylori* Might Predispose the Onset of Atherosclerosis through Indirect Modification of the Microbiota

In this review, the data supporting three different associations are presented: *H. pylori*-atherosclerosis, *H. pylori*-microbiota, and microbiota-atherosclerosis, but the connections between these associations and the mechanisms behind them are not clearly understood. Regarding the *H. pylori*-atherosclerosis association, some possible explanations consider, (a) the bacterial direct action over the arterial walls, (b) indirect induction of endothelial damage by the gastric inflammatory process, (c) cross-reaction between antibodies against the CagA antigen with epitopes on the arterial walls, (d) induction of hypercholesterolemia by inhibition of LDL capture, and (e) foam cell formation by regulation of the reverse cholesterol transport. However, none of these explanations are completely satisfactory. Time is a key factor for the natural history of disease in *H. pylori* infection, and the same circumstance occurs in atherosclerosis and CVDs. In both pathologies, the symptoms appear after a long and silent process that can last even for decades. During the asymptomatic periods, there is a balance between damage and the host response, but in atherosclerosis it is possible to identify early signs or SCA [144, 145]. SCA has been documented in *H. pylori* infected adults without a CVD diagnosis [146–148] and in *H. pylori* infected asymptomatic children [149]. In those children, alterations in the microbiota were documented with a strong association with *Prevotella copri*. The association with *P. copri* is interesting because this bacterium has been associated with obesity, insulin resistance, and type 2 diabetes mellitus, which are risk factors for atherosclerosis [150–153]. However, there are contradictory associations between *P. copri* with health and disease. In a recent publication it was found that *P. copri* shows a diet induced strain-level diversity. This diversity is translated into different metabolic capabilities, and these findings highlight the relevance of knowing the microbial metabolism rather than just describing the bacterial communities [23, 24, 154].

**Figure 1:**
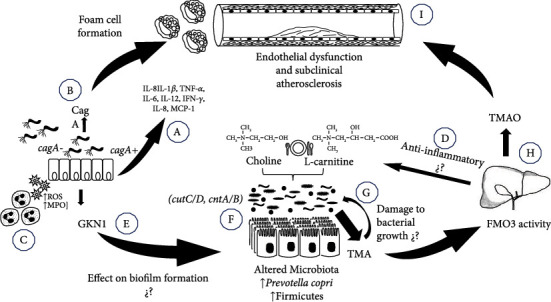
Proposed mechanisms to explain the association between *H. pylori* infection and atherosclerosis. (a) The presence of *H. pylori* induces a systemic inflammatory response that includes the secretion of proinflammatory mediators (IL-1*β*, TNF-*α*, IL-6, IL-12, IFN-*γ*, IL-8, and MCP-1). These mediators may cause a direct damage to vascular endothelial cells as the first step in the induction of atherosclerosis. (b) The direct action of the CagA protein may induce endothelial dysfunction by alteration of reverse cholesterol transport and the foam cell formation. (c) In response to infection, there is an important infiltration of polymorphonuclear cells and macrophages and induction of an oxidative stress state with the release of myeloperoxidase (MPO) and ROS. (d) In response to the oxidative stress, a healing mechanism is activated in the gastric mucosa including the release of different antioxidant enzymes and compounds like L-carnitine. (e) Secretion of GKN1 in the stomach has an effect over the gut microbiota, possibly by its antiamyloidogenic properties altering the biofilm formation. (f) The induced dysbiosis might favor the colonization of bacteria (*Firmicutes*) with the metabolic capability to produce TMA from Choline and L-carnitine. (g) The increased production of TMA may also alter the gut microbiota composition by reducing the iron availability, affecting the growth of other bacterial communities. (h) The increased TMA concentration favors the activity of FMO3 in the liver, resulting in an increased serum levels of TMAO, which is an inductor of endothelial damage. (i) The long-lasting insult into the endothelial cells may result in the development of SCA.

Here, we propose an alternative hypothesis to explain the association between *H. pylori* and atherosclerosis that involves the induction of gut dysbiosis. Our hypothesis is based on the following findings: (1) the *H. pylori* effect over the microbiota composition, (2) the GKN1 and possibly L-carnitine secretion as part of the anti-inflammatory response against the mucosal damage by *H. pylori*, (3) the presence of GKN1 in the gastric mucosa of *H. pylori* infected patients and in the gut of animal models, (4) the induced dysbiosis by GKN1, particularly in the Firmicutes phyla, and (5) the role of the microbiota in the route of TMA/TMAO production, and the TMAO effects on platelets and endothelial cells (Figure 1).

## 5. Potential Research Topics to Explore

The get support for the hypothesis proposed in this review, it is necessary to look for answers of several questions that cannot be addressed in a single project. The following ideas are proposed for the search of answers.

The first controversy that needs to be resolved is whether *H. pylori* infection is, in fact, an additional predisposing factor for atherosclerosis. Different studies recognize the infection as a risk factor after adjusting for other confounding variables. However, as far as we know the *H. pylori* prevalence is different throughout the world, and there is a host genetic component that influences the outcome of the host-parasite relationship. These facts make it necessary to establish an analysis for each region and particular population as it has been suggested for microbiota analyses, in which the virulence of the infecting strains should be considered [155]. In our experience, *H. pylori* infection in Mexico is strongly associated with the presence of SCA and coronary artery disease when compared with a non-CVD control group. However, we still do not know if the presence of the cag pathogenicity island is a relevant factor for this association. [156].

Once an association is established, it is also necessary to analyze whether there are significant differences in the microbiota between individuals with and without *H. pylori*. Given the nature of the disease, three groups should be compared: patients with CVD, healthy individuals but with evidence of SCA, and a *H. pylori* negative group from the open population and without SCA. If there are significant differences in the microbiota between infected and noninfected groups, then it should be investigated whether there are differences in the metabolic capacities for the synthesis of TMA and TMAO plasma levels.

In a second stage, the analysis in a younger population would be mandatory. Most studies have been performed in adult populations, and we know very little about this problem at an early stage. If we assume that an atheroma grows during a long period of time, it becomes relevant to identify changes in the microbiota, coronary artery calcium, TMAO, and GKN1 levels in young adults, teenagers, and children. A longitudinal study would be ideal, but the time and resources to perform this kind of analysis make it very complicated and practically impossible. As an alternative, a transversal study with individuals classified into different age groups could be more feasible.

In a final stage, if there are sufficient evidences that *H. pylori* contributes to atherosclerosis, then it should be evaluated whether its eradication or the microbiota modification represent an alternative preventive therapy. Eradication is a complex decision that will require a cost-benefit analysis. In this regard, the Japanese authorities implemented an early eradication program in 2013, with a consequent reduction in gastric cancer cases [157, 158]. It would be interesting to analyze whether this massive eradication has any effect on the frequency of CVDs. At the end, identifying people at higher risk and taking preventive measures is the main objective of the health authorities worldwide.

In relation to microbiome, the use of microbial metabolites shows encouraging results in animal models. On the one hand, the use of cyclic D, L-*α*-peptides shows reduction of plasma total cholesterol and atherosclerotic plaques, and on the other, the use of 2,3,5,4′-Tetrahydroxy-stilbene-2-O-*β*-D-glucoside (TSG) modifies the overall microbiota and inhibits the atheroma formation [159, 160]. In a similar way, the use of probiotics to inhibit TMA/TMAO concentrations is under study. Some groups of archaea can use methyl compounds (including TMA) as a substrate to produce methane [161]. In particular, the specie *Methanobrevibacter smithii* has shown to decrease the plasma TMAO levels, but all these research areas need further investigation [162].

Another area that needs to be investigated is the role of TMA in bacterial communication. Recently, it was reported that *Streptomyces venezuelae*, a Gram-positive bacterium belonging to the phylum *Actinobacteria* develops a type of growth called “exploratory”. This form of growth consists of an unbranched aerial hypha that can exceed abiotic surfaces in response to unfavorable environmental conditions such as low glucose concentrations or increased pH. This aerial hypha is also induced to other streptomyces species by chemical communication based on volatile organic compounds (VOC). Theses VOC are potential long-term messengers that allow communication between fungi and bacteria over long distances [163]. In the genus *Streptomyces*, the identified VOC is TMA. TMA has also a negative effect on the bacterial growth by reduction of iron availability and alteration of antibiotic resistance [164–166]. So, taking into consideration that the phylum *Actinobacteria*, and the genus *Streptomyces* are part of the the human microbiota, then it is possible to think that TMA may have a role in gut dysbiosis when the environmental conditions are adverse.

## 6. Conclusion

*H. pylori* infection and the microbial metabolite TMAO have been associated with atherosclerosis and CVD. Both infection and elevated TMAO levels are potential factors that may increase the risk for atherosclerosis. However, the mechanisms behind the *H. pylori* induced damage to endothelial tissues are not well understood. In this review, we presented the information regarding the role of microbiota in atherosclerosis as well as the induction of dysbiosis by *H. pylori* infection. Finally, we proposed a hypothesis to explain how *H. pylori* may indirectly induce atherosclerosis by modification of the microbiota. The possibly role of GKN1 in this process, favoring the growth of bacterial groups with the capability to produce TMA and increase the plasma TMAO levels. If this hypothesis is correct, then the interventions to eliminate the bacterium and to restore the gut microbial communities could represent a way to prevent the progression of atherosclerosis and to reduce the incidence of CVD.

## Figures and Tables

**Table 1:** Summary of studies analyzing the relationship between microbiota and CVDs.

Source	Population	Methods	Associated bacterial taxa
Koren et al. [20]	Case-control study *n* = 30 15 patients with atherosclerosis 15 healthy controls	16S rRNA sequencing mouth, stool, and atherosclerotic plaque samples	*Erysipelotrichaceae* and *Lachnospiraceae* families correlate with total cholesterol and LDL cholesterol levels which are atherosclerosis markers
Karlsson et al. [21]	Case-control study *n* = 25 12 patients with atherosclerosis 13 healthy controls	Shotgun sequencing. Stool samples	Atherosclerosis: Genus *Collinsella* Controls: *Roseburia and Eubacterium*
Jie et al. [22]	Case-control study *n* = 405 218 CVD patients 187 healthy controls	Shotgun sequencing. Stool samples	CVD: Higher abundance of *Escherichia coli, Klebsiella spp*., *Enterobacter aerogenes, streptococcus* spp.*, lactobacillus salivarius*, *Solobacterium moorei*, and *Atopobium parvulum*, species. Lower abundance of *Roseburia intestinalis and Faecalibacterium compare prausnitzii Bacteroides spp., Prevotella copri, and Alistipes shahii*
Aryal et al. [25]	Case-control study *n* = 951 478 CVD patients 473 controls	Machine learning strategy Fecal 16S ribosomal RNA sequencing	CVD: *Bacteroides, Subdoligranulum, clostridium, Megasphaera, Eubacterium, Veillonella, Acidaminococcus, and listeria.* Non-CVD: *Faecalibacterium, Ruminococcus, Proteus, Lachnospira, Brevundimonas, Alistipes, and Neisseria*
Asnicar et al. [24]	Clinical trial about the responses to dietary composition *n* = 1098 healthy adults	Shotgun sequencing. Stool samples	Poor health associated species: *Ruthenibacterium lactatiformans, Flavonifractor plautii, Clostridium leptum, Escherichia coli,* *Collinsella intestinalis, clostridium* sp. *CAG:58, Eggerthella lenta,* *Anaerotruncus colihominis, clostridium bolteae, clostridium spiroforme, Ruminococcus gnavus, Clostridium innocuum, Blautia hydrogenotrophica, clostridium symbiosum*, and *clostridium bolteae CAG 59* Health associated species: *F. prausnitzii, E. eligens, Oscillibacter sp. 57 20, Romboutsia ilealis, H. parainfluenzae, Firmicutes bacterium CAG:95, Oscillibacter sp. PC13, V. dispar, Roseburia sp. CAG:182, Veillonella atypica, clostridium sp. CAG:167, V. infantium, B. animalis, P. copri*, and *Firmicutes bacterium CAG:170*
Baragetti et al. [26]	Case-control study *n* = 345 201 with SCA 144 without SCA	16S rRNA and shotgun sequencing. Ultrasound-based analysis for carotid IMT	SCA: *Escherichia,* streptococcus*, Ruminococcus, lactobacillus,* Dorea, Coprococcus, clostridium, Parabacteroides, Eubacterium, and Bifidobacterium Non SCA: *Bacteroides, Ruminococcus*, and *Faecalibacterium prausnitzii*
Chen et al. [23]	Case-control study *n* = 82 31 with CAS 51 without CAS	Shotgun sequencing. Stool samples and ultrasound imaging	CAS: *Eubacteria, Bifidobacterium defectiva, Acidaminococcus intestini,* *Gemella haemolysans, lactobacillus mucosae, Leuconostoc lactis, Megasphaera elsdenii, Ruminococcus sp JC304, streptococcus* *Anginosus, Turicibacter sanguinis*, and *Turicibacter unclassified,* *Escherichia coli, Halomonas unclassified, Klebsiella pneumoniae,* and *Pantoea unclassified.* Non-CAS: *Bacteroides sp 3_1_19, Parabacteroides unclassified*, and *Prevotella copri*

CVD: cardiovascular disease; SCA: subclinical atherosclerosis; CAS: carotid atherosclerosis.

**Table 2:** Summary of studies analyzing the relationship between *H. pylori* and CVDs.

Reference	Samples	Methods	Findings
Mendall et al. [71]	Case-control study *n* = 185 111 coronary heart disease cases and 74 controls	Serology	Coronary heart disease is associated with *H. pylori* seropositivity. OR 2.15 (1-07 to 4 29 95% CI) *P* = 0.03
Franceschi et al. [83]	Umbilical cord and atherosclerotic artery sections	Anti-CagA imunohistochemistry	Anti-CagA antibodies cross-react with arterial wall antigens
Kowalski et al. [77]	46 coronary artery disease cases 19 coronary artery postmortem biopsies	PCR (16S rRNA gene) serology (anti-*H.* *pylori* and anti-CagA)	Identification of *H. pylori* DNA in atherosclerotic plaques
Shmuely et al. [84]	Prospective study of patients examined for presence of aortic atheroma *n* = 188	Transesophageal echocardiography serology (*H. pylori*) western blot (CagA)	The *H pylori* infection, with CagA+ strains, was associated with an increase in the incidence of atherosclerosis
Gunji et al. [72]	Case-sectional survey *n* = 5095	Serology anthropometric and metabolic data	*H. Pylori* infection was significantly associated with metabolic syndrome.
Franceschi et al. [85]	Clinico- pathological study (*n* = 134), and meta-analysis (9 studies)	Anti-CagA serology and immunohistochemistry	CagA seropositivity was significantly associated with the occurrence of acute coronary events. OR 1.34 (95% CI, 1.15–1.58, *P* = 0.0003).
Kędzia et al. [78]	Atherosclerotic plaques from the femoral and iliac arteries (*n* = 51)	*H. Pylori* culture	Positive culture in 3/51 samples
Huang et al. [86]	Coronary heart disease patients infected or not infected with H. pylori *n* = 159	Anti-CagA serology and metabolic data	Coronary atherosclerosis is more severe in patients with *H. pylori* cag + infection (*P* < 0.05)
Park et al. [73]	Case-control study *n* = 2029 1214 H*. p.* positive 815 H*. p.* negative	Serology, Coronary artery calcification score by computed tomography	*H. Pylori* seropositivity is associated to coronary artery calcification score in SCA
Longo-Mbenza et al. [74]	Prospective study (10 years) *n* = 205	Carotid ultrasound (IMT) and *H. Pylori* serology	*H. Pylori* seropositivity was a predictor for the development of atherosclerotic plaque after adjustment for traditional risk Factors. OR 2.3 (95% CI, 1.2-7.2)
Mitra et al. [79]	Deep sequencing and metagenomic analysis in atherosclerotic plaques *n* = 22	Illumina's TruSeq next-generation sequencing and fluorescence in situ hybridization	The most prevalent Species in symptomatic atherosclerotic plaques were *lactobacillus rhamnosus*, *Neisseria polysaccharea*, *helicobacter pylori*, and *Acidovorax spp*.
Liu et al. [167]	Meta-analysis 26 studies involving 21,829 individuals	A literature search and selection of studies comparing the prevalence of *H. pylori* in patients with MI	*H. Pylori* infection is associated with an increased risk of myocardial infarction. OR 1.73 (95% CI, 1.37–2.17, *P* < .00001)
Raut et al. [92]	Cross sectional study of chronic gastritis patients with or without *H. pylori* infection *n* = 80	Chemiluminescence immunoassay for homocysteine and CRP	Elevated homocysteine levels in *H. pylori* gastritis induce endothelial dysfunction and may be the basis of the association between *H. pylori* and coronary artery disease.
Shimoda et al. [87]	Analysis of serum samples from gastric cancer patients and healthy controls	LC-MS/MS and TEM	CagA is present in serum exososmes from patients infected with CagA-positive strains. These vesicles may be responsible to deliver CagA and play a role in the development of extra gastric disorders
Kim et al. [75]	Cross-sectional study including healthy subjects *n* = 37,263	*H. Pylori* serology. Anthropometric and metabolic data	*H. Pylori* infection was a significant and independent risk factor for dyslipidemia Higher LDL-C (RR 1.21; 95% CI, 1.12–1.30; *P* < 0.001) Lower HDL-C (RR 1.10; 95% CI, 1.01–1.18; *P* = 0.021)
Lee et al. [80]	Cross sectional study of healthy subjects *n* = 463	Endoscopy, CLO test computed tomography	Coronary artery calcium scores were significatively higher in *H. Pylori* infected individuals. *H. Pylori* infection is associated with the development of subclinical coronary artery stenosis (OR 2.813, 95% CI, 1.051 ± 7.528, *P* = 0.04)
Choi et al. [76]	Cross sectional study of healthy subjects *n* = 2,251	Serology and cardio-ankle vascular index	Hp-seropositivity was significantly associated with arterial stiffness (OR 1.36; 95% CI 1.10–1.68, *P* = 0.005)
Wang et al. [93]	Meta-analysis 40 case-control and cohort studies *n* = 19,691	Studies that analyze the prevalence of *H. pylori* in patients with adverse cardiovascular events	*H pylori* infection increases the risk of developing atherosclerosis and CVD (OR 1.51, 95% CI 1.34-1.70)
Xia et al. [89]	Case-control study in humans and in a mouse model	Ultrasound assessment in patients and vascular contraction and relaxation in mice	Endothelial dysfunction observed in *H pylori* infection is induced by an exosome-mediated mechanism.
Ninomiya et al. [90]	Drosophila model expressing CagA in the adult eye	A large-scale genetic screen using	Identification of low-density lipoprotein receptor (LDLR) as a novel CagA target for the inhibition of LDL uptake into cells

IMT: intima-media thickness; MI: myocardial infarction; CRP: C-reactive protein; LC- MS/MS: liquid chromatography-tandem mass spectrometry; TEM: transmission electron microscopy.

**Table 3:** Summary of studies analyzing the relationship between *H. pylori* and gut microbiota.

Reference	Samples	Methods	Findings
Ktsoyan et al. [94]	Blood samples 37 FMF patients 14 PU patients 43 healthy controls	Gas chromatography/mass spectrometry	The profile of LCFAs in the human metabolome is disease specific and can potentially be used as a disease marker
Yin et al. [95]	Gerbil model infected with *H. pylori*	Stomach and duodenum bacteria culture	*H. Pylori* infection changes the distribution and abundance of microflora in the stomach and duodenum
Kienesberger et al. [96]	Murin model C57BL/6	16S rRNA gene sequencing	*H. Pylori* infection influences the host microbiota not only in the stomach but also remotely in the intestine
Schulz et al. [97]	Cross sectional study 8 H*. pylori* positive 16 H*. pylori* negative	Duodenal biopsies and aspirates 16S rRNA gene sequencing	*H. Pylori* infection induces changes in the duodenal microbiota Increase in the abundance of proteobacteria
Benavides-Ward et al. [98]	Cross-sectional study *n* = 56 children 28 H*. pylori* positive 28 *H. pylori* negative	*H. Pylori* and 13 genera identified by PCR on feces	There are differences in the microbiota between children with and without *H. pylori*. The presence of *Proteobacteria*, *clostridium*, *Firmicutes*, *and Prevotella* was significantly higher in children with *Helicobacter*
Iino et al. [101]	Cross-sectional study *n* = 884	*H. Pylori* diagnosis by serology and NGS of 16S rRNA gene	*H. Pylori* infection would be associated with lactobacillus in the human gut microbiota
Frost et al. [99]	212 H*. pylori* infected and 212 *H. pylori* Negative subjects	*16S* rRNA gene sequencing	*H. Pylori* infection induce profound alterations and increase the diversity in human fecal microbiota
Wang et al. [103]	Cross-sectional study *n* = 313 128 H. pylori positive 185 H. pylori negative	Shotgun metagenomic sequencing of fecal samples	*H. Pylori* is associated with changes in human intestinal microbiome, particularly with vitamin B12 biosynthesis
Dash et al. [102]	Cross-sectional study *n* = 60 12 *H. pylori* positive 48 H*. pylori* negative	V4 region of 16S rRNA gene sequencing	*H. Pylori* infection increased the abundance of members of *Succinivibrio*, *Coriobacteriaceae, Enterococcaceae*, and *Rikenellaceae*, as well as *Candida glabrata* and other unclassified fungi
White et al. [107]	Case-control study *n* = 35 19 H*. pylori* patients 16 control subjects	16S rRNA gene sequencing	Increased abundance in *H. pylori* subjects: *Atopobium, Gemellaceae, Micrococcaceae, Gemellales and Rothia*
Overstreet et al. [135]	C57BL/6 mouse model WT vs GKN^−/−^	16S rRNA gene sequencing	Gkn1^−/−^ mice exhibited a reduced prevalence of bacteria associated to obesity (*Firmicutes*)

FMF: familial Mediterranean fever; PU: peptic ulceration; LCFAs: long chain fatty acids.

## Data Availability

No data is available.

## Additional Points

*Significance Statement.* Cardiovascular diseases are one of the first causes of death worldwide, and one of their initial steps is the development of atherosclerosis. It is known that *H. pylori* infection and gut dysbiosis are associated with atherosclerosis, but the exact mechanism behind this associations is not completely understood. Recent publications indicate that *H. pylori* gastric colonization can also alter the intestinal microbiota, and in this review, we present the information that support the hypothesis about the effect that *H. pylori* infection exert over the gut microbiota composition to indirectly induce the development of atherosclerosis.
